# Defining molecular glues with a dual-nanobody cannabidiol sensor

**DOI:** 10.1038/s41467-022-28507-1

**Published:** 2022-02-10

**Authors:** Shiyun Cao, Shoukai Kang, Haibin Mao, Jiayu Yao, Liangcai Gu, Ning Zheng

**Affiliations:** 1 Howard Hughes Medical Institute, Department of Pharmacology, University of Washington, Seattle, WA 98195 USA; 2 Department of Biochemistry and Institute for Protein Design, University of Washington, Seattle, WA 98195 USA

**Keywords:** Mechanism of action, X-ray crystallography, Small molecules, Proteins

## Abstract

“Molecular glue” (MG) is a term coined to describe the mechanism of action of the plant hormone auxin and subsequently used to characterize synthetic small molecule protein degraders exemplified by immune-modulatory imide drugs (IMiDs). Prospective development of MGs, however, has been hampered by its elusive definition and thermodynamic characteristics. Here, we report the crystal structure of a dual-nanobody cannabidiol-sensing system, in which the ligand promotes protein-protein interaction in a manner analogous to auxin. Through quantitative analyses, we draw close parallels among the dual-nanobody cannabidiol sensor, the auxin perception complex, and the IMiDs-bound CRL4^CRBN^ E3, which can bind and ubiquitinate “neo-substrates”. All three systems, including the recruitment of IKZF1 and CK1α to CRBN, are characterized by the lack of ligand binding activity in at least one protein partner and an under-appreciated preexisting low micromolar affinity between the two proteinaceous subunits that is enhanced by the ligand to reach the nanomolar range. These two unifying features define MGs as a special class of proximity inducers distinct from bifunctional compounds and can be used as criteria to guide target selection for future rational discovery of MGs.

## Abstract (web summary)

Molecular glue has been used as a broad term describing a class of protein interaction-promoting compounds. Here, the authors outline two unifying thermodynamic features to formally define molecular glues and guide their prospective discovery.

## Introduction

Protein–protein interactions mediate a myriad of biological functions and are susceptible to modulations by small molecules. While extensive efforts in the past have been focused on the development of protein–protein interaction inhibitors as therapeutic agents and research tools^1^, an increasing number of retrospective analyses has indicated that chemical inducers of protein–protein interactions can be found in a variety of proteinaceous systems^2^. The founding members of such chemical inducers are the natural products, cyclosporin A, FK506, and rapamycin, which elicit their immunosuppressive effects by physically coupling immunophilins and calcineurin^3^. Importantly, bifunctional molecules with two distinct chemical moieties connected by a linker can be prospectively developed to induce spatial proximity between two proteins^2,4^. Such a strategy is now widely adopted for developing proteolysis targeting chimeras (PROTACs), which promote targeted protein polyubiquitination and degradation by bridging a preselected target protein to a ubiquitin ligase^5,6^.

Six years after PROTACs was introduced by Deshaies and colleagues in 2001^5^, we raised the concept of “molecular glue” (MG) to explain the mechanism of action of the master plant hormone, auxin, which is distinct from PROTACs and allosteric switches^7^. With a regulatory role in almost every aspect of plant growth and development, auxin promotes the degradation of a family of transcription repressors by enhancing their otherwise weak interactions with the SCF^TIR1^ ubiquitin ligase complex^8^. Instead of allosterically inducing conformational changes in the F-box protein TIR1, auxin fills a gap between the E3 ligase and the degron motif of its substrate, complementing a suboptimal protein–protein interface. Remarkably, the mechanism of action of auxin is recapitulated by the therapeutic compound, thalidomide, and its derivatives, such as lenalidomide and pomalidomide. These compounds, collectively known as immunomodulatory drugs (IMiDs), can promote the degradation of a host of cellular proteins by extending the subpar interfaces they form with CRBN, the substrate receptor of the CUL4-RBX1-DDB1 (CRL4) ubiquitin ligase complex^9–20^. The same MG mechanism has subsequently been revealed for antitumor aryl-sulfonamides, which reprograms another CRL4 substrate receptor, DCAF15, to bind and degrade certain RNA-binding proteins^21–26^. More recently, a panel of CDK12 inhibitors have been shown to act as MGs to enable a neo-morphic interaction between CDK12 and DDB1, inducing CRL4-catalyzed Cyclin K ubiquitination and degradation^27–29^.

Despite the importance of MG compounds in nature and targeted therapeutics, the definition of MG remains elusive. Confusions between MGs and PROTACs in the literature blur the unique properties of MGs and their potential to overcome long-standing challenges in drug discovery against unligandable targets that are intractable to conventional approaches as well as the emerging bifunctional ligand strategy. Here, we report structural and quantitative analyses of a de novo engineered dual-nanobody cannabidiol sensor, in which the ligand acts as an MG to induce protein–protein interaction. By comparing it with the auxin and IMiDs systems, we outline the common thermodynamic characteristics of the three systems that differentiate MGs from other type of proximity inducers.

## Results

### Overall structure of a CBD-bound dual-nanobody sensor

**Fig. 1:**
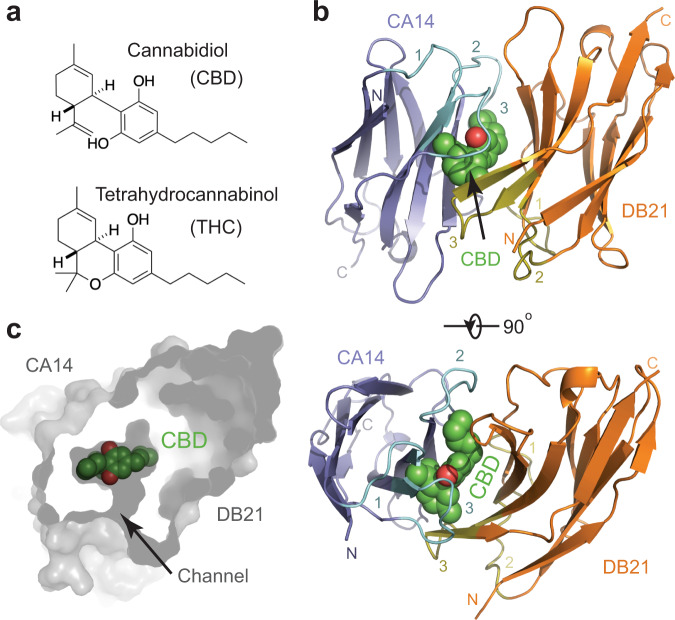
Overall structure of a CBD-bound dual-nanobody sensor. **a** Chemical structures of cannabidiol (CBD) and tetrahydrocannabinol (THC). **b** Two orthogonal views of the crystal structure of the CA14-CBD-DB21 ternary complex. CBD is shown as green and red spheres. CA14 and DB21 are shown as cartoon diagrams and colored blue and orange, respectively. Three complementarity determining regions (CDRs) of CA14 and DB21 are highlighted in cyan and olive, respectively. CDR1, CDR2, and CDR3 are labeled as 1, 2, and 3. **c** Cross-section view of the CBD-binding pocket formed by the nanobody dimer. CBD, shown in green and red spheres, is located in the central pocket with a nearby channel too small for it to enter or escape.

Cannabidiol (CBD), which has recently been approved by the FDA for the treatment of epilepsy, is a non-psychotropic phytocannabinoid with multiple pharmacological effects and broad therapeutic potential^30^ (Fig. 1a). A combinatorial binders-enabled selection method has recently been developed to construct a highly specific CBD sensor, which consists of two nanobodies that would only heterodimerize in the presence of the ligand^31^. In this method, an “anchor binder” nanobody was first selected out of a combinatorial phage-displayed nanobody library using biotinylated CBD as a bait. A “dimerization binder” nanobody was subsequently identified based on its affinity toward the CBD-bound, but not the apo, form of the anchor binder. In order to reveal the structural basis of such a CBD-sensing system, we determined the crystal structure of a ternary complex formed by an anchor binder (CA14), CBD, and a matching dimerization binder (DB21) at 2.0 Å resolution (Fig. 1b, Supplementary Fig. 1a, and Supplementary Table 1).

In the crystal, CA14 and DB21 share a nearly identical scaffold, which is expected for their common framework sequence (Supplementary Fig. 1a, b). The CBD ligand is sandwiched between the two nanobodies, which pack against each other in a face-to-face and largely antiparallel manner (Fig. 1b). Consistent with their monomeric nature and ligand-dependent interaction, the two nanobodies are engaged with each other in a spatial orientation that is distinct from the classic VH-VL heterodimers^32–34^ (Supplementary Fig. 2a). In the CA14-CBD-DB21 ternary complex, each of the three molecules makes extensive and direct contacts with the other two partners. Overall, the complex buries a total of ~1400 Å^2^ surface area among all three components with about two-thirds contributed by protein–protein interactions. As an inducer of protein–protein interaction, CBD is completely sequestered at the center of the nanobody dimer and snuggly fits into the central pocket constructed by the two immunoglobin β-barrels. Although a small channel can be found connecting the central pocket to the solvent, it is too narrow for CBD to enter or escape without the dissociation of the two nanobodies (Fig. 1c and Supplementary Fig. 1c).

### Intermolecular interfaces among CBD, CA14, and DB21

**Fig. 2:**
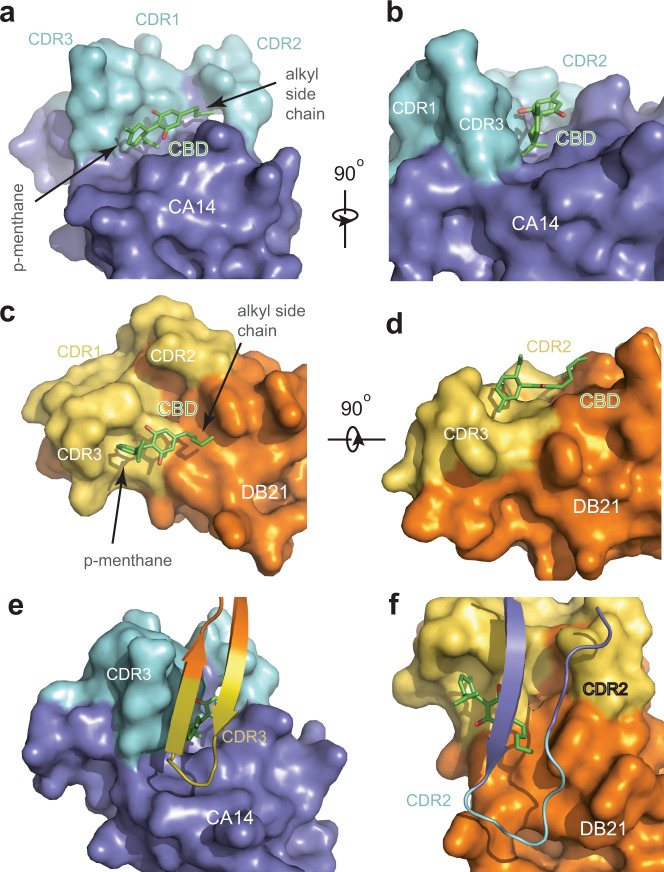
Intermolecular interfaces among CBD, CA14, and DB21. **a**, **b** Two orthogonal close-up views of the interface between CBD and CA14. CBD is shown in green and red sticks. CA14 is shown in dark blue surface representation except for three complementarity determining regions (CDRs) highlighted in cyan. **c**, **d** Two orthogonal close-up views of the interface between CBD and DB21. DB21 is shown in orange surface representation except for three CDRs highlighted in yellow. **e**, **f** Two close-up views of the interface between CA14 and DB21 with CBD sandwiched in the middle. A CDR and its nearby region of a nanobody (shown in cartoon model) directly pack against their equivalent parts in the other nanobody (shown in surface representation).

As expected for its ability to bind CBD independent of DB21, CA14 is characterized by a surface groove formed among the three complementarity determining regions (CDRs) and the framework of the nanobody (Fig. 2a, b). In an extended conformation, CBD is embedded in this overall hydrophobic groove with its five-carbon alkyl side chain secured by CDR2 at one end and its *p*-menthane moiety vertically seated next to CDR3 at the other. Its central resorcinol ring is stabilized by a hydrogen bond formed between one of its two hydroxyl groups and the side chain of Asp34 in CDR1 (Supplementary Fig. 2b). The CA14 nanobody was originally selected against tetrahydrocannabinol (THC), a psychoactive CBD analog with an essentially identical molecular weight (Fig. 1a). Such a high selectivity can be rationalized by the overall perpendicular orientation between the *p*-menthane and resorcinol rings of CBD bound to the anchor binder (Fig. 2b), which is incompatible with the closure of the oxygen bridge found in THC.

In contrast to CA14, the CBD-contacting surface of DB21 is noticeably flat with only a minor depression created by CDR3 that accommodates the methyl group sticking out from the *p*-menthane moiety of the ligand (Fig. 2c, d). The rest of CBD simply makes van der Waal contact with the framework of the dimerization nanobody. Surrounding these unremarkable interactions that DB21 makes with CBD, the dimerization nanobody uses multiple structural elements including fifteen residues to form interfaces with the anchor binder (Supplementary Fig. 3). At one end of the CBD-binding pocket, CDR3 of DB21 and its nearby framework residues directly pack against their equivalent parts in CA14, thereby completely burying the p-menthane moiety of the ligand (Fig. 2e and Supplementary Figs. 1a, 2b). At the opposite end, DB21 uses the CDR2 loop and its flanking sequences to seal off the CBD-binding site by interacting with their corresponding regions in the CA14 nanobody (Fig. 2f). Collectively, the multiple interfaces formed between the ligand and each of the two nanobodies and between the two nanobodies themselves appear to cooperatively stabilize the ternary complex.

### Quantitative analysis of CBD dual-nanobody sensor

**Fig. 3:**
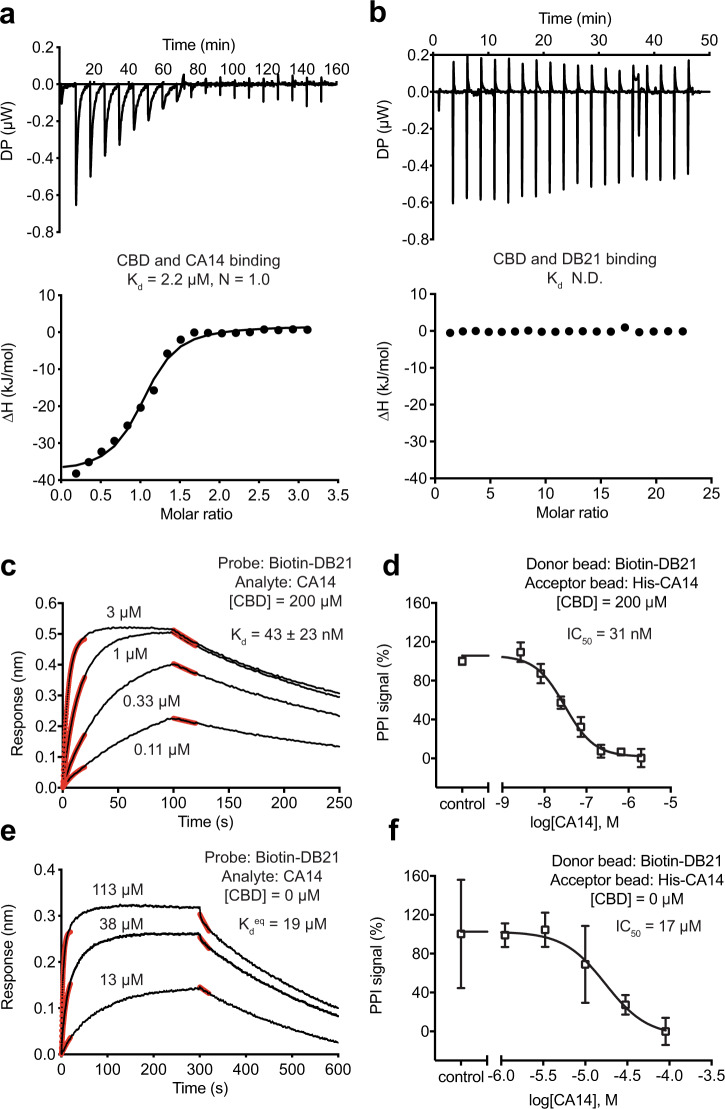
Quantitative analysis of CBD dual-nanobody sensor. **a**, **b** CBD binding to CA14 and DB21 measured by isothermal titration calorimetry (ITC). Source data are provided as a Source Data file. **c**, **e** BioLayer interferometry (BLI) measurements of CA14 and DB21 interaction in the presence and absence of CBD at a saturating concentration. Black lines represent the kinetics of association and dissociation of CA14 with biotinylated DB21 immobilized on the probe. Red lines represent curve fitting at the initial phase of association and dissociation. For CA14–DB21 interaction without CBD, there was a discrepancy between the *K*_d_ values determined by kinetics (3.0 ± 0.8 μM) and equilibrium measurements. The latter is consistent with the result obtained in AlphaScreen assays. *K*_d_, dissociation constant. Source data are provided as a Source Data file. **d**, **f** AlphaScreen competition assays for assessing DB21 binding to CA14 with and without CBD. Data were presented as mean ± s.d. of *n* = 3 biologically independent samples. IC_50_, half-maximum inhibitory concentration. Source data are provided as a Source Data file.

To obtain a better understanding of the dual-nanobody CBD-sensing system, we next quantitatively dissected the intermolecular interactions among its three components. Using isothermal calorimetry (ITC), we confirmed that the anchor nanobody CA14 alone can bind CBD with an affinity in the single-digit μM range (2.2 μM), as previously reported (6 μM) (Fig. 3a). By contrast, isolated DB21 showed no detectable affinity toward the ligand (Fig. 3b). This result is in agreement with the DB21-CBD interface revealed in the ternary complex structure, which is predominantly flat. Using BioLayer Interferometry (BLI), we next measured the affinity between DB21 and CBD-bound CA14. Consistent with the extensive and continuous interface formed between DB21 and CBD-occupied CA14, the dimerization and anchor nanobodies can form a tight complex with a dissociation constant of 43 nM in the presence of CBD at a saturating concentration (Fig. 3c). This high affinity was further confirmed by an AlphaScreen-based competition assay, in which label-free CA14 was used to compete for complex formation between affinity-tagged CA14 and DB21 immobilized on the Alpha beads (Fig. 3d). Interestingly, although DB21 was originally selected against CBD-free CA14, we were able to detect a relatively weak interaction between the two nanobodies with a quantifiable affinity of around 20 μM using the BLI and AlphaScreen-based competition assays (Fig. 3e, f). CBD, therefore, strengthens the protein–protein interaction between the two nanobodies by ~450-folds.

### The critical role of CBD-DB21 interface

The structure of the CA14-CBD-DB21 complex suggests that CBD might promote CA14–DB21 interactions via two possible mechanisms. CBD could stabilize the conformation of the CDRs of CA14 in a form that is compatible for binding DB21. CBD could also enhance CA14–DB21 interaction by re-surfacing CA14 and extending the protein–protein interaction interface. Despite our extensive efforts, CA14 remains refractory to crystallization. We, therefore, set to assess the contribution of the CBD-DB21 interface to the ternary complex formation by structure-guided mutagenesis analysis.

**Fig. 4:**
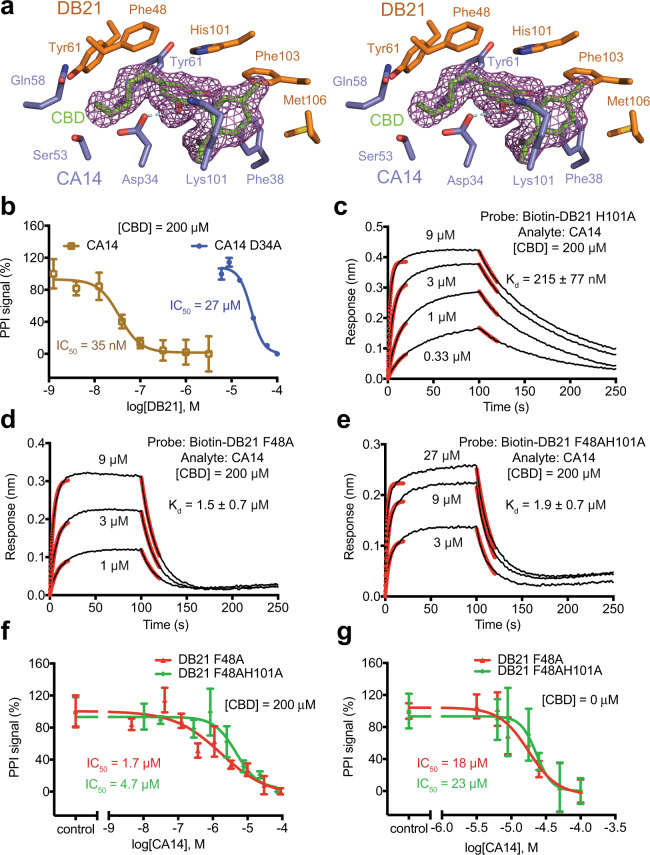
Mutational analysis of CBD dual-nanobody sensor. **a** A close-up stereo view of CA14 and DB21 residues interacting with CBD (green and red sticks) with its positive *F*_*o*_ − *F*_*c*_ electron density contoured at 1σ (purple mesh). CA14 (blue) and DB21 (orange) residues are shown in the stick model. The hydrogen bond between CA14 Asp34 and CBD are represented by cyan dashes. **b** AlphaScreen competition assays for assessing the effect of D34A mutation in CA14 (WT CA14 in brown and CA14 D34A mutant in blue) on ternary complex formation with CBD at a saturating concentration. Data were presented as mean ± s.d. of *n* = 3 biologically independent samples. IC_50_, half-maximum inhibitory concentration. Source data are provided as a Source Data file. **c**–**e** BLI analyses for assessing the effects of H101A, F48A, and F48A-H101A mutations in DB21 on ternary complex formation with CBD at a saturating concentration. Red lines represent curve fitting at the initial phase of association and dissociation. *K*_d_, dissociation constant. Source data are provided as a Source Data file. **f**, **g** AlphaScreen competition assays for assessing the effects of F48A (red) and F48A-H101A (green) mutations on CA14–DB21 interaction in the presence or absence of CBD at a saturating concentration. Data were presented as mean ± s.d. of *n* = 3 biologically independent samples. IC_50_, half-maximum inhibitory concentration. Source data are provided as a Source Data file.

We first validated the crystal structure by introducing an alanine mutation to CA14 Asp34. This residue forms the only hydrogen bond with the ligand and makes one of several polar interactions with DB21 (Fig. 4a and Supplementary Figs. 2b, 3). As expected, such a mutation in CA14 completely disabled the dual-nanobody system to respond to CBD and had a minor impact on the intrinsic affinity between the two nanobodies (Fig. 4b). We next focused on two amino acids in DB21, Phe48, and His101, whose side chains make direct contact with CBD but not CA14 (Fig. 4a). Remarkably, while alanine mutation of His101 reduced the affinity of DB21 towards CBD-bound CA14 by approximately fivefolds, mutation of Phe48, either alone or together with H101A, markedly compromised the ability of the ligand to promote CA14–DB21 interaction (Fig. 4c–g). Instead of an ~450-folds enhancement, CBD could only increase the affinity of CA14 towards the two F48A-bearing DB21 mutants by apptoximately tenfolds. Consistent with the location of these two residues away from the protein–protein interface, their mutations had little effect on the intrinsic affinity between CA14 and DB21 (Fig. 4g). These results indicate that the complementary interface between CBD and DB21 serves as a major determinant of the CBD activity in fostering protein–protein interaction, even though CBD does not show any measurable affinity towards DB21 in the absence of CA14. While these data do not rule out the possibility that CBD binding might optimize the conformation of CA14 CDRs for interacting with DB21, such an effect is unlikely the predominant driver for ternary complex formation stabilized by the ligand.

### Comparison to the auxin-sensing system

The overall topology and intermolecular interactions of the dual-nanobody CBD sensor is highly reminiscent of the auxin receptor system, in which the plant hormone is recognized by a surface pocket of the ubiquitin ligase TIR1 and is covered by the degron motif of TIR1 substrate proteins, Aux/IAAs. Analogous to DB21, the Aux/IAA degron does not feature a hormone-binding site but instead makes direct contacts with both the hormone and the ubiquitin ligase. In doing so, the Aux/IAA degron buries auxin inside its solvent-free pocket. Instead of allosterically inducing conformational changes in its ubiquitin ligase receptor, auxin promotes strong E3-substrate interactions by completing its central interface.

**Fig. 5:**
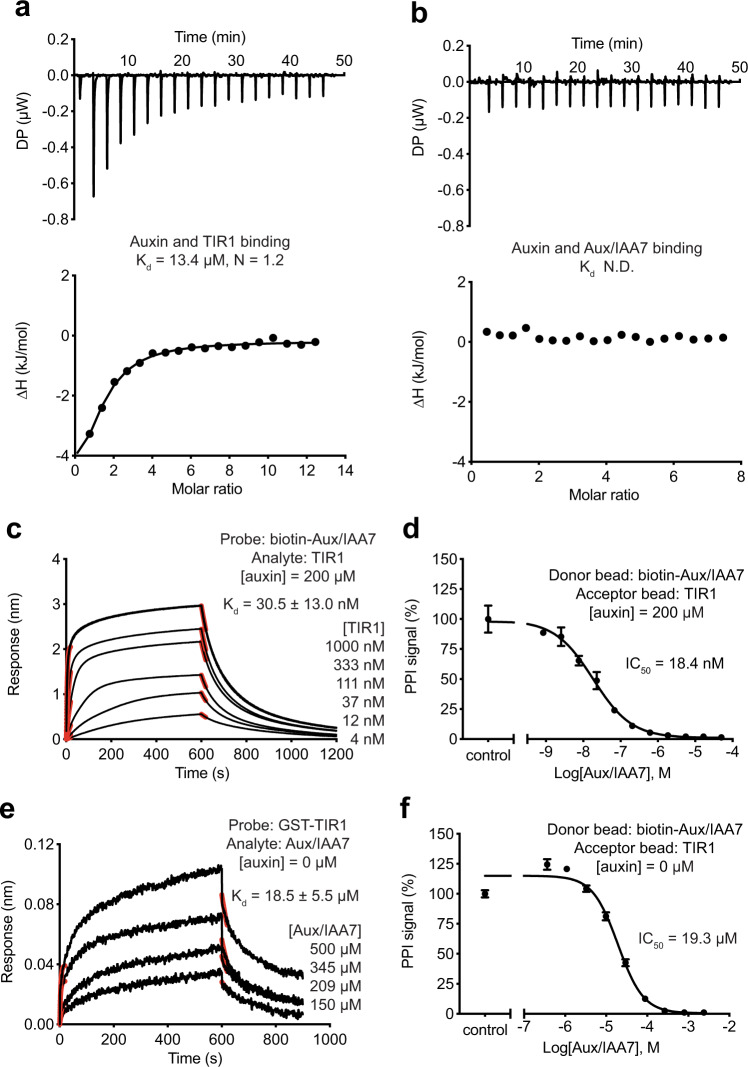
Quantitative analysis of the auxin-sensing system. **a**, **b** Auxin binding to TIR1 and Aux/IAA7 peptide measured by ITC experiments. ND not detected. Source data are provided as a Source Data file. **c**, **d** BLI and AlphaScreen competition assays for assessing the affinity of Aux/IAA7 to TIR1 in the presence of auxin at a saturating concentration. Red lines represent curve fitting at the initial phase of association and dissociation. *K*_d_, dissociation constant. IC_50_, half-maximum inhibitory concentration. Data were presented as mean ± s.d. of *n* = 3 biologically independent samples. Source data are provided as a Source Data file. **e**, **f** BLI and AlphaScreen competition assays for assessing the intrinsic affinity of Aux/IAA7 to TIR1. Red lines represent curve fitting at the initial phase of association and dissociation. *K*_d_, dissociation constant. IC_50_, half-maximum inhibitory concentration. Data were presented as mean ± s.d. of *n* = 4 biologically independent samples. Source data are provided as a Source Data file.

To make a quantitative comparison between the CBD- and auxin-sensing systems, we employed the same biophysical methods to quantify the intermolecular interactions of the auxin receptor complex. Our ITC analysis showed that TIR1 on its own can bind auxin with a 13.4 μM affinity, whereas the free Aux/IAA degron peptide displayed no detectable affinity towards the plant hormone (Fig. 5a, b). In the presence of saturating amount of auxin, the TIR1 E3 ligase and its substrate degron motif strongly interact with each other with an affinity of 30.5 nM as measured by BLI (Fig. 5c), which was further confirmed by the AlphaScreen-based competition assay (Fig. 5d). Remarkably, just like in the case of the dual-nanobody CBD sensor, we were able to reliably detect a weak but quantifiable affinity between the TIR1 E3 ligase and its substrate degron motif in the absence of auxin at ~18–20 μM using both BLI and AlphaScreen assays (Fig. 5e, f). The plant hormone, therefore, is able to enhance the affinity between the TIR1 E3 ligase and the Aux/IAA degron peptide by ~600-folds.

Based on our structural and quantitative analyses, the dual-nanobody CBD sensor is strikingly similar to the ubiquitin ligase-based auxin receptor system in multiple aspects. First of all, in both cases, one of the two protein binding partners (CA14 or TIR1) features a surface pocket that can capture the ligand with a moderate affinity. Second, the ligand in neither case shows any detectable affinity toward the second binding partner (DB21 or Aux/IAA), which lacks a ligand-binding pocket but nonetheless makes direct contact with the ligand. Third, in both systems, each component of the ternary complex forms an interface with the other two components. The protein–protein interface, in particular, is characterized by a quantifiable low micromolar affinity between the two protein components. The ternary complex, therefore, is stabilized by both small molecule-protein interfaces and protein–protein interactions. Fourth, despite the moderate or low affinity between any pair of the three components, the ternary complex is nucleated by a nM affinity between the two protein binding partners enabled by the ligand. Overall speaking, these properties equate the two systems and qualify CBD as an auxin-like MG in promoting the heterodimerization of the two sensor nanobodies.

### Intrinsic affinity between CRBN and neo-substrates

**Fig. 6:**
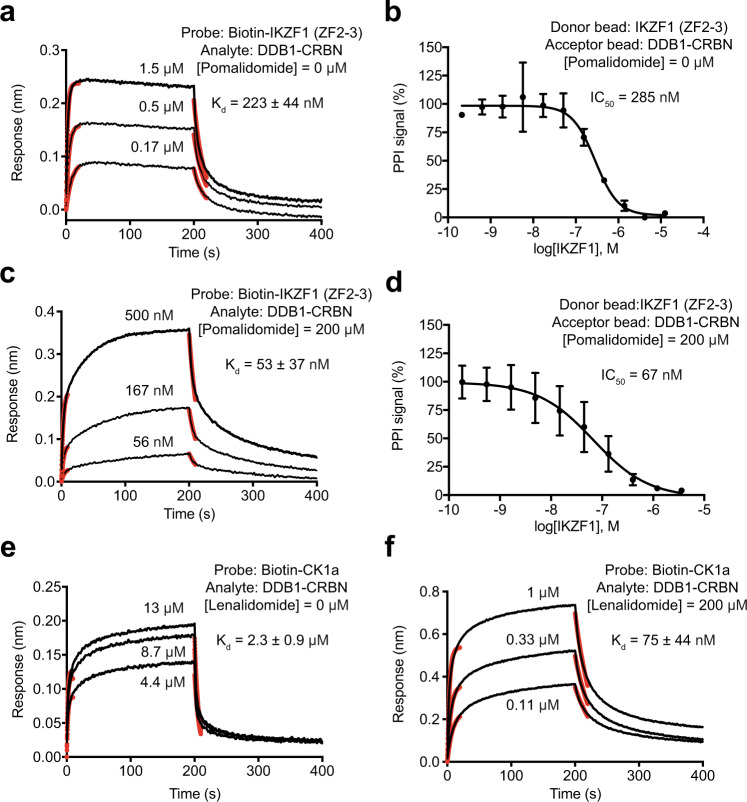
Quantitative analysis of IMiDs system. **a**, **b** BLI and AlphaScreen competition assays for assessing the intrinsic affinity of IKZF1 (ZF2-3) to DDB1–CRBN. Red lines represent curve fitting at the initial phase of association and dissociation. *K*_d_, dissociation constant. IC_50_, half-maximum inhibitory concentration. Data were presented as mean ± s.d. of *n* = 3 biologically independent samples. Source data are provided as a Source Data file. **c**, **d** BLI and AlphaScreen competition assays for assessing the affinity of IKZF1 (ZF2-3) to DDB1–CRBN in the presence of pomalidomide at a saturating concentration. Red lines represent curve fitting at the initial phase of association and dissociation. *K*_d_, dissociation constant. IC_50_, half-maximum inhibitory concentration. Data were presented as mean ± s.d. of *n* = 3 biologically independent samples. Source data are provided as a Source Data file. **e**, **f** BLI measurements of CK1a and DDB1–CRBN interaction in the absence and presence of lenalidomide at a saturating concentration. Red lines represent curve fitting at the initial phase of association and dissociation. *K*_d_, dissociation constant. Source data are provided as a Source Data file.

Similar to CBD and auxin, IMiDs represent a group of prototypical MG compounds distinct from other chemical inducers of proximity by their remarkable chemical simplicity. Previous studies have shown that IMiDs can occupy a tryptophan cage binding site on CRBN with an affinity of ~150–250 nM^35^. Without a ligand-binding pocket, classic neo-substrates of IMiDs-bound CRBN, such as IKZF1 and CK1α, are not expected to have an affinity towards the compounds^12,15^. Despite extensive mechanistic studies of IMiDs, the intrinsic affinity between ligand-free CRBN and its neo-substrates remains elusive. In light of the measurable low μM affinity between the ligand-free protein components of the CBD and auxin systems, we set out to determine whether CRBN has any detectable affinity towards IKZF1 and CK1α. To our surprise, IKZF1 displayed a robust affinity at 200–300 nM toward CRBN in complex with full-length DDB1, as measured in both BLI and AlphaScreen competition assays (Fig. 6a, b). Pomalidomide enhanced their interaction by about fourfolds, reaching 50–70 nM (Fig. 6c, d). Similarly, we determined the affinity between CRBN and CK1α in the absence of an IMiD compound and obtained a *K*_d_ value of 2.3 μM (Fig. 6e). In the presence of saturating amount of lenalidomide, the affinity increases to a *K*_d_ value of 75 nM (Fig. 6f). Therefore, the “neo-morphic” interactions between the DDB1–CRBN E3 ligase and its two classic neo-substrates are actually built upon their considerable intrinsic affinities, which are not high enough to trigger ubiquitination.

The common thermodynamic characteristics shared among the CBD, auxin, and IMiDs systems prompt us to propose that MGs can be defined as a special class of proximity inducers by two fundamental features. First, an MG does not have to have any detectable affinity towards at least one of the two protein partners. Second, the mechanism of action of an MG compound requires direct protein–protein interaction, which is manifested by a quantifiable intrinsic affinity between the two proteinaceous components in the absence of the ligand. In fact, these two properties are two sides of the same coin—the lack of affinity of an MG to one of the binding partners necessitates direct protein–protein contact to stabilize the ternary complex.

### A mathematical model for the auxin-like MG system

**Fig. 7:**
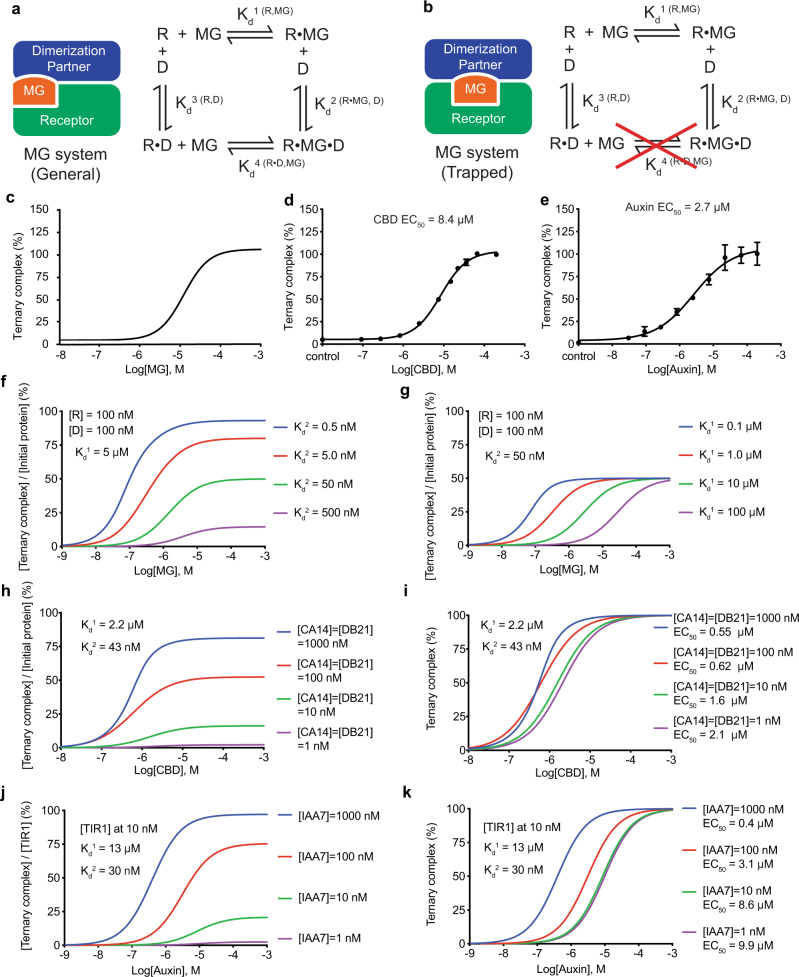
Mathematical simulation of MG system. **a**, **b** Topology and equilibria of a general and topologically trapped MG system. For the topologically trapped MG system, MG (orange) is buried at the protein interface and cannot enter its binding site if the binary protein complex between the Receptor (green) and the dimerization partner (blue) is formed first. R receptor, D dimerization partner, R•MG the receptor-MG binary complex, R•D the binary complex of receptor-dimerization partner, and R•MG•D the ternary complex of receptor-MG-dimerization partner. Four equilibrium dissociation constants, *K*_d_^1 (R,MG)^, *K*_d_^2 (R•MG,D)^, *K*_d_^3 (R,D)^, and *K*_d_^4 (R•D,MG)^, represent the affinity between R and MG, R•MG and D, R and D, and R•D and MG, respectively. **c** MG dose-response curve calculated by a mathematical model. **d**, **e** Experimentally detected MG dose-response curve for CBD and auxin, respectively. Data were presented as mean ± s.d. of *n* = 3 biologically independent samples. EC_50_, half-maximum response concentration. Source data are provided as a Source Data file. **f** Simulation of MG dose-response curve with fixed total protein concentrations [R], [D], and *K*_d_^1^ and variable *K*_d_^2^. Blue, red, green, and purple lines indicate dose-response curves with an increasing *K*_d_^2^ value. **g** Simulation of MG dose-response curve with fixed total protein concentrations [R], [D], and *K*_d_^2^ and variable *K*_d_^1^. Blue, red, green, and purple lines indicate dose-response curves with an increasing *K*_d_^1^ value. **h**, **i** Simulation of CBD dose-response curve with variable total protein concentrations [CA14] and [DB21]. Ternary complex (%) represents [CA14•CBD•DB21]/[CA14•CBD•DB21]_MAX_. Blue, red, green, and purple lines indicate dose-response curves with a decreasing identical [CA14] and [DB21] value. **j**, **k** Simulation of auxin dose-response curve with fixed total [TIR1] and variable [IAA7]. Ternary complex (%) represents [TIR1•auxin•IAA7]/[TIR1•auxin•IAA7]_MAX_. Blue, red, green, and purple lines indicate dose-response curves with a decreasing [IAA7] value.

Given the special properties of MGs, the thermodynamic cycle for ternary complex formation mediated by such compounds is distinct from that of bifunctional proximity inducers, which has been previously described^36^. The three-body equilibria are characterized by two intermediate binary complexes, in which the MG receptor (R) is either bound to the compound (MG) or the dimerization protein partner (D) (Fig. 7a). The complete cycle involves four equilibrium dissociation constants, *K*_d_^1(R,MG)^, *K*_d_^2(R•MG,D)^, *K*_d_^3 (R,D)^, and *K*_d_^4 (R•D,MG)^, which reflect the affinity between R and MG, D and MG-occupied R, R and D, and D-bound R and MG, respectively. While these three-body equilibria describe, in theory, two separate pathways for ternary complex formation, the topology of the auxin- and CBD-sensing systems revealed in their crystal structures strongly suggests that only one pathway can be taken in solution (Fig. 7b). Because the binding site of auxin and CBD is completely buried at the protein interface, these compounds would not be able to enter their binding sites if the binary protein complex is formed first. Auxin and CBD, therefore, represent a class of topologically trapped MGs.

In the ternary complex equilibria depicted in Fig. 7b, if the total concentrations of R and D (R_0_ and D_0_) are both far below *K*_d_^3^, the concentration of MG-free R•D complex will be minimal and negligible (Supplementary Note 1). Omission of the R•D complex formation further simplifies the equilibria and allows us to derive a mathematical model relating the concentration of the ternary complex ([R•MG•D]) to the total concentrations of its three molecular components (R_0_, MG_0_, and D_0_), the affinity of MG towards its receptor *K*_d_^1^, and the affinity between the MG-occupied receptor and the dimerization partner *K*_d_^2^. The resulting quartic equation has only one real solution, which can be used to establish a dose-response curve of MG for ternary complex formation (Supplementary Note 1 and Source Data). When plotted on a logarithmic scale, the MG dose-response curve has a familiar sigmoid shape (Fig. 7c), which is in stark contrast to the bell-shaped dose-response curve produced by the bifunctional proximity inducers^36,37^. The absence of a “hook effect” is consistent with the lack of affinity between an MG compound and the dimerization partner. To validate our mathematical model, we used the AlphaScreen assay to experimentally determine a dose-response curve for both auxin and CBD with fixed total concentrations of the protein components far below their ligand-independent intrinsic affinities. Consistent with the predictions made by our model, both compounds yielded a sigmoid dose-response curve (Fig. 7d, e).

Similar to many conventional drugs, the dose-response curve of MGs is characterized by two critical features, maximal efficacy ([R•MG•D]_MAX_) and potency (EC_50_). These two parameters can be expressed as a function of R_0_, D_0_, *K*_d_^1^, and *K*_d_^2^ (Supplementary Note 1). We note that the maximal efficacy, i.e., the maximal concentration of the ternary complex formed, is determined by the total amounts of the MG receptor and the dimerization partner, as well as the affinity of the dimerization partner to the MG-loaded receptor (*K*_d_^2^), but not the affinity of the MG towards its receptor (*K*_d_^1^) (Fig. 7f–h, j and Supplementary Note 1). The potency of MG (EC_50_), on the other hand, is driven by both *K*_d_^1^ and *K*_d_^2^ in addition to the total concentrations of the protein components (Fig. 7f, g and Supplementary Note 1). When *K*_d_^1^ » *K*_d_^2^, R_0_, and D_0_, which is true in our AlphaScreen assay, *K*_d_^1^ determines the upper limit of EC_50_. Interestingly, with a rising value of R_0_ and D_0_, either individually or together, EC_50_ descends from *K*_d_^1^ owing to the slower *k*_off_ resulting from the trapping of the MG compound by the dimerization partner (Fig. 7i, k and Supplementary Fig. 4a, b).

### Cooperativity of MGs

In previous studies of bifunctional proximity inducers, a cooperativity term (represented by α) has been used to characterize the effects of protein–protein interaction in ternary complex formation^36,37^. It is defined as the ratio of the dissociation constants for the interactions between the ligand and one of the two protein components in the absence and presence of the other. In the case of MGs, cooperativity (α = *K*_d_^3^/ *K*_d_^2^) is a central parameter describing the activity of the compounds. Although protein complex formation in the absence of an MG might not be always easy to quantify due to the relatively weak affinity, *K*_d_^3^ is critical for the assessment of cooperativity, which informs the competence of an MG compound. Based on our quantitative measurements, auxin and CBD confer cooperativity of ~600 and ~450 to the auxin- and CBD-sensing systems, respectively (Table 1). Even higher cooperativity is achieved by the rationally developed NRX-252114 compound, which is capable of enhancing the binding between β-TrCP and a hypophosphorylated β-catenin degron (pSer33/Ala37) peptide by 680-folds^38^. Of note, the cooperativity of an MG system is physically determined by the complementary interface between the ligand and the dimerization partner, which we have experimentally characterized for CBD. Defects in this interface increase *K*_d_^2^ but spare *K*_d_^3^, thereby compromising cooperativity (Fig. 4d–g). By contrast, the cooperativity of a bifunctional proximity inducer system is attributable to the ligand-induced protein–protein interface^37^. While bifunctional compounds can act with a negative, zero, or positive cooperativity, MG molecules rely on positive cooperativity to function.

**Table 1:** Thermodynamic features of select molecular glues.

	Auxin	CBD	NRX-252114*	Pomalidomide	Lenalidomide	E7820**
R	TIR1	CA14	β-TrCP	DDB1–CRBN	DDB1–CRBN	DCAF15
D	IAA7 degron	DB21	β-catenin degron (pSer33/S37A)	IKZF1(ZF2-3)	CK1α	RBM39 (R1R2)
*K*_d_^3 (R,D)^ (µM)	18.5	19.0	0.272	0.223	2.3	4.6
*K*_d_^2 (R•MG,D)^ (nM)	30.5	43	0.4	53	75	160
Cooperativity α	607	442	680	4.2	28	29
M.W. (Da)	175	314	510	273	259	336

*R* receptor, *D* dimerization partner, *MW* molecular weight, Da dalton, *K*_d_^3 (R,D)^ dissociation constant of the MG-free R-D complex, *K*_d_^2 (R•MG,D)^ dissociation constant of the MG-bound R-D complex.

*Ref. ^38^

**Ref. ^22^

## Discussion

The term “molecular glue” has been used since the 1980s to describe proteins with bridging functions^39,40^. In 1992, Schreiber and Crabtree used it once on cyclosporin and FK506, which are cyclic peptide immunosuppressants^3^. Its usage on small molecules, however, had remained invisible to keyword search until we independently coined it to describe the mechanism of action of auxin^7^. We chose to use this specific term to not only contrast auxin with other hormones acting as allosteric switches, but also distinguish it from the concept of PROTACs proposed by Deshaies and Crews^5^. In this study, we formally define “molecular glue” as a unique class of protein interaction-promoting compounds, which extend the existing interaction interface without showing a detectable affinity toward (at least) one of the binding partners. Our definition not only emphasizes the non-detectable affinity between the MG compound and one of the macromolecular partners but also highlights the quantifiable dissociation constant between the ligand-free MG receptor and the dimerization partner. The former property distinguishes MG from the bifunctional molecules, while the latter parameter manifests the direct protein–protein interface, which enables MGs to promote ternary complex formation despite lacking any affinity towards the dimerization partner.

Importantly, our formal treatment of “molecular glue” is disparate from its usage as a general term for chemical inducers of proximity^41^. Heralded by PROTACs in targeted protein degradation, the prospective discovery of chemical inducers of proximity is entering the mainstream of small molecule drug discovery. Although MGs and bifunctional compounds such as PROTACs both belong to the larger category of chemical inducers of proximity, they are distinguished by their distinct dose-response curves and potential targets. In targeted protein degradation, MGs are particularly capable of inducing the ubiquitination and degradation of non-ligandable substrates, including natively disordered polypeptides and proteins without a druggable pocket. None of the substrate protein surfaces in direct contact with MG compounds, such as auxin, IMiDs, indisulam, and NRX-252114, is amendable to ligand binding in the absence of the E3 ligase. The substrates destabilized by these compounds, therefore, are intractable to the PROTACs approach. Such a distinction underscores the importance of their separate classification and calls for totally different strategies for MG discovery.

Among the prototypical MG systems, the intrinsic affinities between TIR1 and the degron of its natural substrates, AUX/IAAs, and particularly between CRBN and its two neo-substrates, CK1α and IKZF1, have been underappreciated. Their measurable values, nevertheless, are consistent with the previously documented observations—in the absence of IMiDs, IKZF1 has been identified as a CRBN binding protein by proteomics studies and CK1α can be destabilized by CRBN in response to Wnt^42,43^; recombinant IAA7, the substrate of TIR1, can also pull down the residual amount of the F-box protein without auxin^44^. Moreover, their weak interactions are echoed by the low μM affinities recently reported for the DCAF15-RBM39 and DDB1-CDK12-Cyclin K interacting partners^22,29^ as well as the bipartite degron of JAZ1 that docks to the F-box protein COI1 via an α-helix next to the binding pocket of jasmonate-isoleucine, another MG plant hormone^45^. Thus, most, if not all, simple MG compounds act on proteins that already have a propensity to interact with each other. Therefore, future efforts in the prospective discovery of MGs should first focus on targeting protein pairs that have a detectable affinity. In targeted protein degradation, for example, higher priority shall be placed on a ubiquitin ligase that has a measurable weak affinity towards a preselected neo-substrate with high therapeutic potential for developing MG protein degraders. The question remains as to what constitutes the affinity range of weak protein interactions that are amendable to the action of drug-like diffusible MG compounds. Conceivably, with increasing chemical complexity or taking advantage of avidity, MG compounds might be able to act on protein partners that have very weak intrinsic affinities^46^. However, when such an intrinsic affinity becomes extremely low or none, a bifunctional molecule will be required to bridge the partners if both are ligandable.

Taking advantage of the trapped topology of the two MG compounds in this study, we have developed a mathematical model that allows users to evaluate the impact of the total concentrations of proteins and compounds as well as two characteristic dissociation constants, *K*_d_^1 (R,MG)^ and *K*_d_^2 (R•MG,D)^, on ternary complex formation. Importantly, the same model can be applied to a non-topologically trapped MG system, if the affinity between the compound-free protein components, *K*_d_^3 (R,D)^, is much weaker than the affinity of the MG to the receptor, *K*_d_^1 (R,MG)^. Regardless of the ternary complex topology, our study underscores the importance of the experimental determination of *K*_d_^3 (R,D)^, which is necessary to determine the cooperativity of the MG-mediated protein–protein interaction. As a hallmark of the MG action, cooperativity stems from the interface between the MG and the unligandable protein partner. It represents a critical property guiding any effort in optimizing an MG compound.

As a class of topologically trapped MGs, auxin and CBD use their entire surface to enhance protein–protein interactions in the two compound-sensing systems. On one side, the two compounds bind to their receptors (TIR1 and CA14) with a moderate affinity (*K*_d_^1 (R,MG)^). On the other side, the ligands use the rest of their surface to complement the dimerization partners. These properties enable the two compounds to mediate protein complex formation with high efficiency, despite a relatively small molecular weight. Such a higher glue efficiency is expected to improve the drug-likeness of MG compounds, which is particularly relevant for CNS-targeting therapeutic agents. Our mathematical modeling suggests that the potency of auxin and CBD in promoting ternary complex formation is dictated by *K*_d_^1 (R,MG)^, *K*_d_^2 (R•MG,D)^, and the level of the protein partners. On the one hand, a moderate affinity of an MG towards its receptor will prevent the compound from blocking the receptor when the dimerization partner is absent, a scenario that is relevant to targeted protein degradation. On the other hand, such a feature of an MG might limit the potency of the compound when the levels of both protein partners are low.

Outside targeted protein degradation, retrospective studies of MGs have begun to showcase their versatile and promising power in inactivating enzymes by stabilizing autoinhibitory inter-domain interactions^47^, potentiating enzymatic activities by promoting protein oligomerization^48,49^, blocking protein–protein interaction by inducing unnatural homodimerization^50^, modulating the functions of client proteins by targeting their common adapter^51^, and even modifying splicing by promoting RNA-U1 snRNP interaction^52^. In some cases, the MG compounds act on naturally interacting partners. In other cases, MGs are found to promote “neo-morphic” interactions. We postulate that these MG-enhanceable unnatural interactions are widespread in nature and reflect the general tendency of proteins to undergo nonspecific interaction that are nevertheless partner selective. In such cases, MG compounds, in essence, convert nonspecific and nonproductive weak protein interactions into specific and productive binding. With a clear definition of MGs and a quantitative understanding of their actions, rational strategies can now be developed to expedite their prospective discovery.

## Methods

### Protein preparation

Nanobodies (CA14, DB21, and their mutants) were constructed with an N-terminal pelB-His-tag for periplasmic expression in *E.coli*. To release proteins from the periplasm by osmotic shock, the resuspended pellets were added with 30 mL of TES/4 buffer (1:4 dilution of the TES buffer containing 0.2 M Tris-HCl pH 8.0, 0.5 mM EDTA, 0.5 M sucrose) and gently shaken on ice for 45 min. His-tagged nanobodies were purified by Ni-NTA resin with subsequent TEV protease treatment to remove the His-tag. Tag-free nanobodies were further purified by size exclusion chromatography. Purified CA14, DB21, and CBD with molar ratio 1: 1: 1.2 were incubated at room temperature for 2 h to assemble the ternary complex and concentrated to 10–20 mg/ml for crystallization. To obtain the biotinylated DB21 for affinity measurements, an additional Avi-tag was designed after the TEV cleavage site. After Ni-NTA affinity purification and TEV cleavage of His-tag, Avi-DB21 was generated and subsequently biotinylated by biotin ligase BirA-catalyzed biotinylation reaction. Biotin-DB21 was further purified by size exclusion chromatography to remove BirA and extra biotin. *Arabidopsis thaliana* TIR1-ASK1 (GST-tagged or tag-free) was co-expressed and purified from insect cells using glutathione affinity chromatography followed by TEV cleavage. The complex was further purified by anion exchange and size exclusion chromatography. Non-labeled and biotinylated IAA7 degron peptides were synthesized by GenScript. Human IKZF1^ZF2-3^ (141–196) was expressed with an N-terminal GST-TEV-Avi-tag in Hi5 monolayer insect cells. IKZF1^ZF2-3^ was isolated from soluble cell lysate by glutathione affinity chromatography. After cleavage by TEV, the Avi-IKZF1^ZF2-3^ was generated and further purified by size exclusion chromatography. Human CK1α was expressed with an N-terminal His-Set3-TEV-Avi-tag in Hi5 monolayer insect cells. CK1α was isolated from soluble cell lysate by Ni-NTA affinity purification. After cleavage by TEV, Avi-CK1α was further purified by cation exchange and size exclusion chromatography. To label Avi-IKZF1^ZF2-3^ and Avi-CK1α with biotin, BirA-catalyzed biotinylation reactions were performed. Biotin-labeled IKZF1^ZF2-3^ and CK1a were subsequently purified by size exclusion chromatography. Human CRBN^∆1-40^ and full-length DDB1 were fused with an N-terminal SUMO-tag and His-Msb-tag, respectively, and co-expressed in Hi5 monolayer insect cells. CRBN–DDB1 complex was isolated from soluble cell lysate by Ni-NTA affinity purification. After cleavage by TEV, the tag-free CRBN–DDB1 complex was further purified by anion exchange and size exclusion chromatography.

### Crystallization, data collection, and structural determination

Crystals of the CA14-CBD-DB21 complex were grown at 25 °C by the hanging-drop vapor diffusion method with 0.1 μL protein samples mixed with an equal volume of reservoir solution (0.2 M Ammonium citrate tribasic, pH 7.0, 0.1 M imidazole, pH 7.0 and 20% polyethylene glycol monomethyl ether 2000). The largest crystal was harvested and flash-frozen in the crystallization buffer supplemented with 20% glycerol at −170 °C. The X-ray diffraction data set was collected at the BL8.2.1 beamline at the Advanced Light Source in Berkeley and was integrated and scaled by HKL2000 package^53^. The complex structure was solved by molecular replacement using the program Phaser-MR of PHENIX and two nanobodies structural models predicted from their protein sequences by Phyre2 web portal as search templates^54^. The complex structure model was rebuilt, refined and ligand-fitted using COOT^55^ and PHENIX^56^. PyMOL (The PyMOL Molecular Graphics System, Version 2.0 Schrödinger, LLC) and LIGPLOT^57^ were used to generate figures.

### Isothermal titration calorimetry (ITC)

ITC experiments were performed at 25 °C using a MicroCal PEAQ-ITC (Malvern). CBD at 960 or 750 µM in the syringe was titrated into 8 µM DB21 or 45 µM CA14 in the microcalorimeter cell, respectively. About 1 mM auxin in the syringe was titrated into 25 µM IAA7 or 15 µM TIR1 in the microcalorimeter cell, respectively. A total of 18 (2 µL) injections were added whilst stirring at 750 rpm. The data were analyzed with the MicroCal PEAQ-ITC Analysis Software. All ITC experiments have been repeated at least two times.

### BioLayer interferometry (BLI)

Binding affinity between dimerization partner and receptor in the absence or presence of saturating amount of MG was measured using the Octet Red 96 (ForteBio, Pall Life Sciences) following the manufacturer’s procedures. The optical probes were coated with streptavidin, loaded with 200 nM biotinylated proteins (DB21, IAA7, IKZF1, or CK1α), or anti-GST probes loaded with 200 nM GST-tagged TIR1. Subsequently, the probes were quenched with 0.5 mM biocytin or 1 µM His-GST prior to kinetic binding analysis. The reactions were carried out in black 96 well plates maintained at 30 °C. The reaction volume was 200 μL in each well. The binding buffer contained 25 mM HEPES, pH 7.4, 100 mM NaCl, 1 mM TCEP (omitted in CA14–DB21 binding), 0.1% Tween-20, and 0.05 mg/mL bovine serum albumin. As the analyte, tag-free protein (CA14, TIR1, DDB1–CRBN, or IAA7) was tested at various concentrations. To detect the affinity between the dimerization partner and MG-bound receptor, 200 µM MG (CBD, auxin, lenalidomide, or pomalidomide) was added into the analyte to saturate receptor proteins (CA14, TIR1, or DDB1–CRBN). There was no binding of the analyte to the unloaded probes. Binding kinetics of the analyte at different concentrations were measured simultaneously. The data were analyzed by the Octet data analysis software. The initial 10–20 s of association and dissociation curves were fit with a 1:1 binding model. The *k*_on_ and *k*_dis_ values were used to calculate the dissociation constant (*K*_d_) with kinetic analysis of direct binding. In all measurements, except for Fig. 3e, the *K*_d_ value determined from the steady-state equilibrium measurements is similar to the *K*_d_ value calculated from the kinetic measurements. All BLI experiments have been repeated at least three times.

### Amplified luminescence proximity homogeneous assay

AlphaScreen assays for measuring protein–protein interactions were performed using an EnSpire reader (PerkinElmer). Receptor protein (His-CA14, GST-TIR1, or His-DDB1–CRBN) was bound to AlphaScreen acceptor beads. Biotinylated dimerization partner (DB21, IAA7 degron peptide, or IKZF1^ZF2-3^) was immobilized to streptavidin-coated AlphaScreen donor beads. The donor and acceptor beads were brought into proximity by the interactions between receptor protein and its corresponding dimerization partner in the absence and presence of saturating amount of MG (CBD, auxin, or pomalidomide). Excitation of the donor beads by a laser beam of 680 nm promotes the formation of singlet oxygen. When an acceptor bead is in close proximity, the singlet oxygen reacts with thioxene derivatives in the acceptor beads and causes the emission of 520–620 nm photons, which are detected as the binding signal. If the beads are not in close proximity to each other, the oxygen will return to its ground state and the acceptor beads will not emit light. In our hands, AlphaScreen displays a higher dynamic range than time-resolved fluorescence energy transfer (TR-FRET). Competition assays were performed by titrating label-free proteins (CA14, DB21, IAA7 degron peptide, or IKZF1^ZF2-3^) at various concentrations. To establish the binding signal, the concentration of tagged receptor proteins and biotinylated dimerization partners were first titrated with a fixed amount of donor and acceptor beads in the matrix. The concentration of each component was selected based on the rising AlphaScreen signal before the beads became saturated. In the absence of CBD, 200 nM His-CA14 and 67 nM biotinylated DB21 (wild type or mutant) were used. In the presence of CBD, 22 nM His-CA14 and 7.4 nM biotinylated DB21; 67 nM His-CA14 and 22 nM biotinylated DB21^F48A^; 200 nM His-CA14 and 22 nM biotinylated DB21^F48AH101A^; 200 nM His-CA14^D34A^ and 67 nM biotinylated DB21 were used respectively. For the auxin system, the competition experiments were conducted with 0.9 nM of GST-TIR1 and 8.3 nM biotinylated IAA7 degron peptide in the absence of auxin or 0.3 nM of GST-TIR1 and 2.8 nM biotinylated IAA7 degron peptide in the presence of 200 µM auxin. For the IMiDs system, 0.9 nM of His-DDB1–CRBN and 8.3 nM biotinylated IKZF1^ZF2-3^ were used in the assays without and with 200 µM pomalidomide. The binding assay buffer contained 25 mM HEPES, pH 7.4, 100 mM NaCl, 1 mM TCEP (omitted in samples containing CA14 and DB21), 0.1% Tween-20, and 0.05 mg/mL Bovine Serum Albumin. About 5–10 μg/mL donor and acceptor beads were used in the assays. The experiments were performed in three or four replicates. IC_50_ was determined using nonlinear curve fitting of the dose-response curves generated with Prism 7 (GraphPad). AlphaScreen assays were also used to measure the MG (CBD or auxin) dose-response curve of inducing ternary complex formation. The experiments were conducted with 22 nM His-CA14 and 7.4 nM biotinylated DB21 or 0.3 nM GST-TIR1 and 2.8 nM biotinylated IAA7 degron peptide. CBD or auxin were titrated at various concentrations to induce the interactions between receptor (CA14 or TIR1) and dimerization partner (DB21 or IAA7). The experiments were done in triplicates. MG dose-response curves were generated with Prism 7 (GraphPad) and EC_50_ values were determined by using nonlinear curve fitting. All AlphaScreen experiments have been repeated at least three times.

### Reporting Summary

Further information on research design is available in the Nature Research Reporting Summary linked to this article.

## Supplementary information

Supplementary Information

Reporting Summary

## Data Availability

Structural coordinates are deposited in the Protein Data Bank with accession code 7TE8. Source data are provided with this paper.

## Source data

Source Data
