# Supplementary material for: Defining molecular glues with a dual-nanobody cannabidiol sensor
Source: Nat Commun. 2022 Feb 10;13:815. doi: 10.1038/s41467-022-28507-1 (PMC8831599; doi:10.1038/s41467-022-28507-1)
Supplement: Supplementary file 1 — Supplementary Information [file 41467_2022_28507_MOESM1_ESM.pdf]

Supplementary Information

Supplement to: “Defining Molecular Glues with A Dual-Nanobody Cannabidiol Sensor”

Contents **Page**

Supplementary Table 1	2
Supplementary Figure 1	3
Supplementary Figure 2	4
Supplementary Figure 3	5
Supplementary Figure 4	6
Supplementary Note 1	7

Supplementary Table 1. Data collection and refinement statistics

	CA14-CBD-DB21 ternary complex
**Data collection**	
Space group	P 2 2_1 2_1
Cell dimensions	
a, *b*, *c* (Å)	62.7, 69.2, 109.1
α, β, γ (°)	90, 90, 90
Resolution (Å)	41.15 – 2.00 (2.07 – 2.00) *
R_{merge}	0.125 (0.541)
$I / \sigma I$	57.4 (2.2)
Completeness (%)	99.8 (98.6)
Redundancy	7.1 (6.8)
CC1/2	0.996 (0.948)
**Refinement**	
Resolution (Å)	50.00 -2.00
No. reflections	32798
R_{work} / R_{free}	0.181 / 0.211
No. atoms	4083
Protein	3708
Ligand/ion	46
Water	329
B-factors	29.49
Protein	29.19
Ligand/ion	20.56
Water	34.15
R.m.s. deviations	
Bond lengths (Å)	0.009
Bond angles (°)	1.06

Note: A single crystal was used for diffraction data collection and structure determination. *Values in parentheses are for highest-resolution shell.

Supplementary Figure 1. Comparison of CA14 and DB21 nanobodies and surface representation of the ternary complex. a, Alignment and secondary structure assignments of DB21 and CA14. Three complementarity determining regions (CDRs) are highlighted in red with residues involved in ligand-protein and protein-protein interactions indicated. **b,** Structure alignment of DB21 and CA14. Structures are shown in Cα tracing diagrams. **c,** Three views of the surface representation of the CA14-CBD-DB21 complex. CA14 and DB21 are coloured in blue and orange, respectively. The three CDRs of CA14 and DB21are highlighted in cyan and olive, respectively. CBD shown in green and red spheres is trapped in the central pocket of the nanobody dimer with a nearby channel too small for CBD to enter or escape.

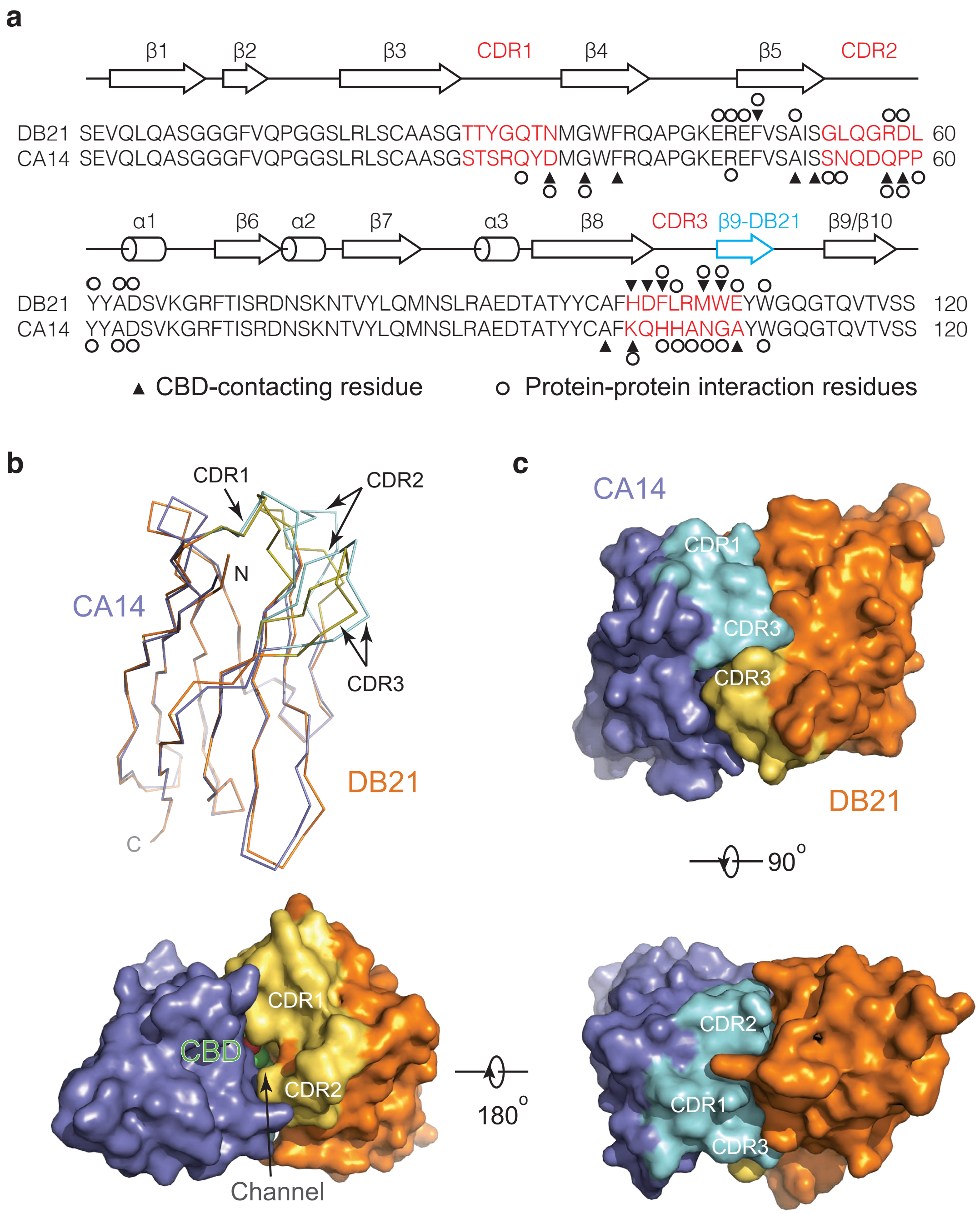

Supplementary Figure 2. CBD-nanobody interactions. a, Structural comparison of the catalytic antibody IF7 in complex with a transition state analogue (TSA) (PDB: 1FIG) and the CA14-CBD-DB21 complex. Ligand (TSA or CBD) is shown in stick model, while proteins are shown as cartoon diagrams. **b,** Interactions between CBD and surrounding DB21 and CA14 residues. The 2D scheme of ligand interactions is generated by LIGPLOT.

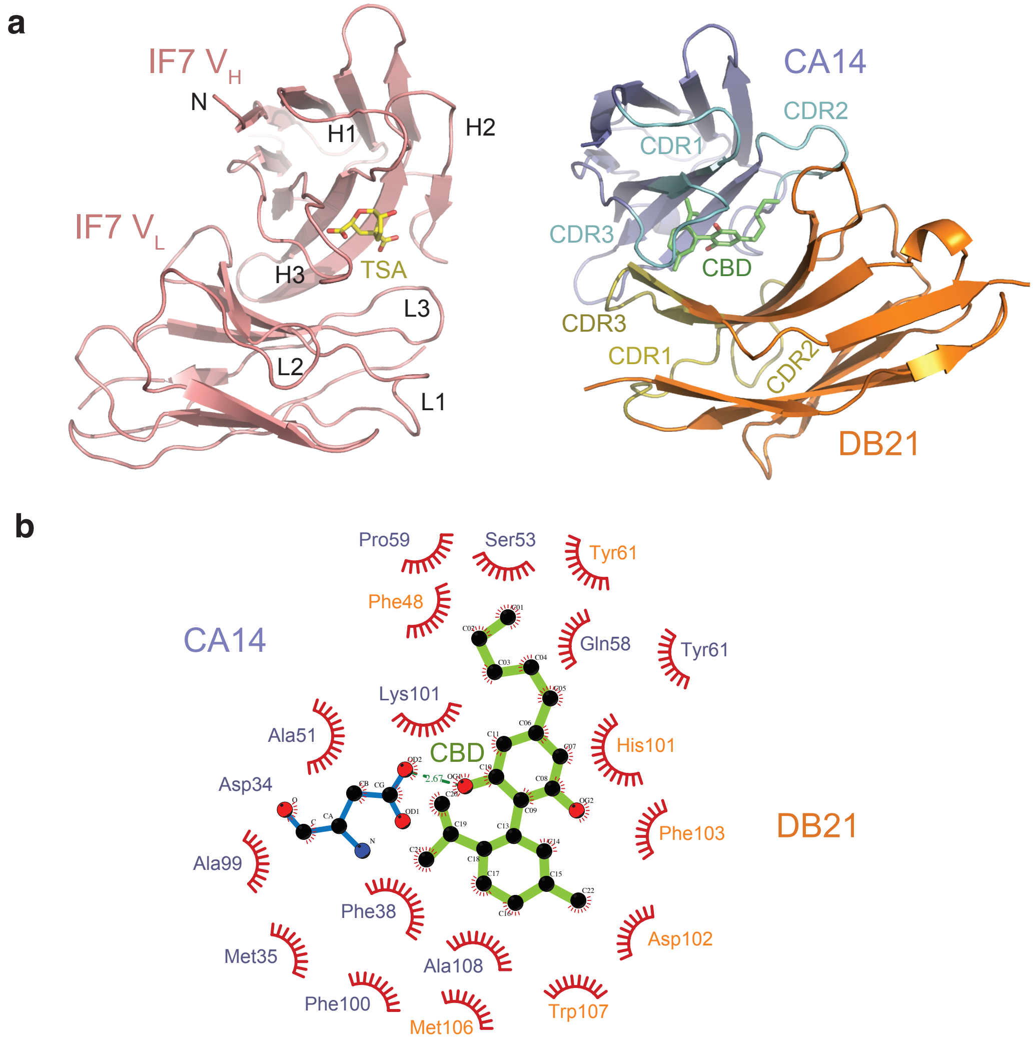

Supplementary Figure 3. CA14 and DB21 interactions. The hydrophobic and polar (green dashes) interactions between CA14 and DB21 residues. The 2D scheme of interactions is generated by LIGPLOT.

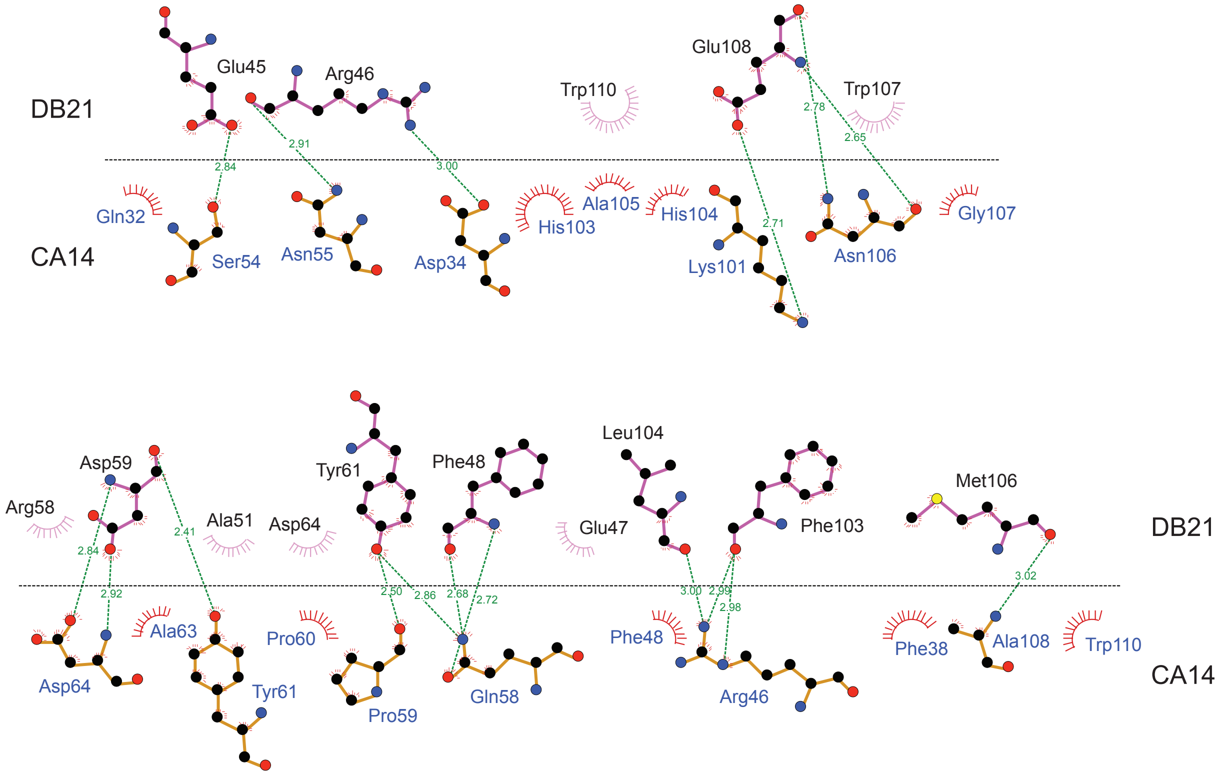

Supplementary Figure 4. a, b, Mathematical simulation of auxin dose-response curve with variable total concentrations of [TIR1] and [IAA7]. Y-axis in panel b: ternary complex (%) represents [TIR1•auxin•IAA7] / [TIR1•auxin•IAA7]$_{MAX}$.

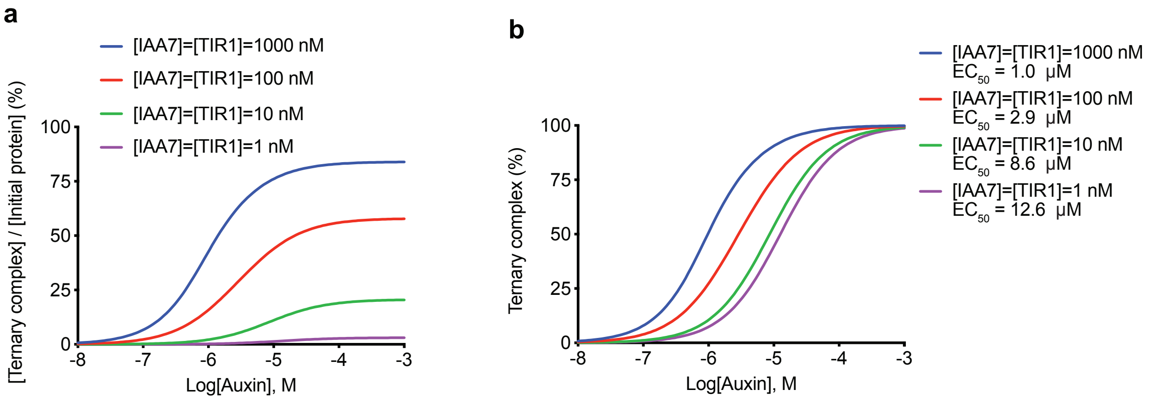

Supplementary Note 1. Mathematical model of a topologically trapped MG system.

Initial total concentrations of receptor, dimerization partner and MG are defined as R_0, D_0 and MG_0, respectively. The equilibrium dissociation constant (K_d)-based Equation (1) and (2) are used to derive Equation (3), which is a quartic equation in a standard format as $aY^4+bY^3+cY^2+dY+e=0$ (coefficients a=1, while b, c, d and e are expressed in MG_0, D_0, R_0, K_d^1 and K_d^2). Y is [R•MG•D] at equilibrium and can be calculated by solving Equation (3). [R•MG•D] reaches maximal (Y_{MAX}) when MG is titrated to a concentration high enough to saturate all receptor proteins, i.e., R_0 = [R•MG] + [R•MG•D] ($R_0 = X + Y_{MAX}$). Thus, Equation (2) can be written as $K_d^2 = (R_0 - Y_{MAX})(D_0 - Y_{MAX}) / Y_{MAX}$, which is a quadratic function. The solution of Y_{MAX} is shown in Equation (4). EC_{50} is the concentration of MG at which $Y = 0.5Y_{MAX}$. Equation (2) can be written as $K_d^2 = X(D_0 - 0.5Y_{MAX}) / 0.5Y_{MAX}$, which could be rearranged as $X = 0.5Y_{MAX} K_d^2 / (D_0 - 0.5 Y_{MAX})$. Substituting X in Equation (1) with $0.5Y_{MAX} K_d^2 / (D_0 - 0.5 Y_{MAX})$ yields EC_{50} as shown in Equation (5).

At equilibrium

R_0 = [R] + [R•D] + [R•MG] + [R•MG•D]
D_0 = [D] + [R•D] + [R•MG•D]
MG_0 = [MG] + [R•MG] + [R•MG•D]

When $R_0 \ll K_d^3$ & $D_0 \ll K_d^3$
[R•D] $\ll R_0$, D_0
$R_0 \approx$ [R] + [R•MG] + [R•MG•D]
$D_0 \approx$ [D] + [R•MG•D]

Let [R•MG] = X & [R•MG•D] = Y

$$K_d^1 = \frac{(R_0 - X - Y)(MG_0 - X - Y)}{X} \quad (1)$$

$$K_d^2 = \frac{X(D_0 - Y)}{Y} \quad (2)$$

Rearrange equation (2) and substitute X in equation (1) with $YK_d^2 / (D_0 - Y)$

$$Y^4 - (MG_0 + R_0 + 2D_0 + 2K_d^2)\,Y^3$$
$$+[(MG_0 + R_0)\,D_0 + (R_0 + D_0 + K_d^2)(MG_0 + D_0 + K_d^2) + K_d^1 K_d^2]\,Y^2$$
$$-[(R_0 + D_0 + K_d^2)\,MG_0 D_0 + (MG_0 + D_0 + K_d^2)\,R_0 D_0 + K_d^1 K_d^2 D_0]\,Y + MG_0 R_0 D_0^2 = 0 \quad (3)$$

$$Y_{max} = \frac{R_0 + D_0 + K_d^2 - \sqrt{(R_0 + D_0 + K_d^2)^2 - 4R_0 D_0}}{2} \quad (4)$$

$$EC_{50} = 0.5Y_{max} + \frac{0.5\,Y_{max} K_d^2}{D_0 - 0.5\,Y_{max}} + \frac{0.5\,Y_{max} K_d^2 K_d^1}{(R_0 - 0.5\,Y_{max})(D_0 - 0.5\,Y_{max}) - 0.5\,Y_{max} K_d^2} \quad (5)$$
